# Optimale Sauerstoffversorgung – neue Erkenntnisse aus der COVID-19-Therapie?

**DOI:** 10.1007/s00063-022-00914-8

**Published:** 2022-04-14

**Authors:** Daniel Dankl

**Affiliations:** Universitätsklinik für Anästhesiologie, perioperative Medizin und allgemeine Intensivmedizin, Uniklinikum Salzburg, Müllner Hauptstraße 48, 5020 Salzburg, Deutschland

**Keywords:** Sauerstoffverbrauch, Hypoxie, Laktat, Sauerstoffsättigung, Physiologie, Oxygen consumption, Hypoxia, Lactates, Oxygen saturation, Physiology

## Abstract

Für die Energiegewinnung und damit Überlebensfähigkeit menschlicher Zellen ist Sauerstoff von essenzieller Bedeutung. Kommt es durch Krankheit, Verletzung oder Veränderung der Umweltfaktoren zu einer Störung der Sauerstoffversorgung, ist der menschliche Körper bis zu einem gewissen Grad in der Lage, Kompensationsmechanismen in Gang zu setzten, um trotzdem ein ausreichendes Sauerstoffangebot für die Funktion und Integrität der Zellen bzw. Organsysteme breitzustellen. Werden diese Kompensationsmechanismen ausgeschöpft oder überlastet, droht ein Funktionsausfall von Zellen und Organsystemen. Im klinischen Alltag ist es bei abnormen Sauerstoffwerten oft schwierig zu entscheiden, ob die körpereigenen Kompensationsmechanismen noch ausreichen oder ob invasivere Therapieoptionen mit entsprechenden Nebenwirkungen angewandt werden sollten, um Organschäden zu verhindern. Um dies entscheiden zu können, ist neben der Kenntnis der aktuellen Studienlage und der Zielparameter ein Verständnis der grundlegenden physiologischen Mechanismen der Sauerstoffversorgung der Zellen notwendig. Ziel dieser Übersicht ist es, die physiologischen Grundlagen des Sauerstoffangebots zu wiederholen, aufzuzeigen, wie ein unzureichendes Sauerstoffangebot erkannt werden kann, und die aktuelle Studienlage bzw. die Leitlinien zu Sauerstoffzielwerten zu beleuchten. Zwar hat die Pandemie durch die Coronaviruserkrankung 2019 (COVID-19) die Aufmerksamkeit für Pathophysiologie und Therapiestrategien bei Oxygenierungsstörungen rezent in den Fokus gerückt, allerdings haben sich aus dieser Pandemie kaum neue Erkenntnisse hinsichtlich der Sauerstoffzielwerte ergeben. Somit bleiben die bisher empfohlenen Sauerstoffzielwerte unverändert bestehen.

## Abstract (en)

Oxygen is essential for energy production and thus for the survival of human cells. If oxygen delivery is disrupted due to illness, injury or changes in environmental factors, the human body is to a certain extent able to activate compensatory mechanisms to ensure adequate delivery of oxygen for the function and integrity of the cells and organ systems. If these compensatory mechanisms are exhausted or overloaded, there is a risk of functional failure of cells and organ systems. In clinical practice, it is often difficult to decide whether the body’s own compensation mechanisms are still sufficient or whether more invasive therapy options and their side effects should be used to prevent organ damage. The aim of this review is to reiterate the basic physiological mechanisms of oxygen delivery to cells, to show how insufficient oxygen can be detected, and to highlight the current state of studies and guidelines on target oxygen levels. Although the coronavirus disease 2019 (COVID-19) pandemic has brought recent attention to the pathophysiology and therapeutic strategies of oxygenation disorders, little new knowledge regarding oxygen targets has emerged from this pandemic. Thus, the previously recommended oxygen target values remain unchanged.

## Hintergrund

Müssen Organsysteme in der Intensivmedizin unterstützt oder ersetzt werden, stellt sich immer die Frage nach Zielwerten, die durch die Therapie erreicht werden sollen. Wie bei der Therapie anderer Organsysteme kommt es auch bei der Organunterstützungstherapie bzw. beim Organersatz der Lunge mit zunehmender Invasivität der Maßnahmen zu relevanten Nebenwirkungen, sodass die (Über‑)Therapie die Prognose möglicherweise per se verschlechtert. In diesem Zusammenhang ist insbesondere die Frage nach dem optimalen bzw. ausreichenden Sauerstoffgehalt im Blut von essenzieller Bedeutung. Eine Definition der in der Sauerstoffversorgung relevanten Begriffe gibt Tab. 1.

**Table 1:** 

Begriff	Definition
S_p_O_2_	Perkutan photometrisch gemessene Sauerstoffsättigung des funktionellen Hämoglobins
S_a_O_2_	Die durch ein Blutgasanalysegerät bestimmte Sauerstoffsättigung des Hämoglobins im arteriellen Blut
p_a_O_2_	Partialdruck von physikalisch gelöstem Sauerstoff im arteriellen Blut
Hypoxämie	Sauerstoffsättigung des Hämoglobins liegt unter dem Normalbereich (S_a_O_2_ normal > 94 %)
Hypoxie	Unzureichende Versorgung mit Sauerstoff im Verhältnis zum Sauerstoffbedarf
Hyperoxämie	p_a_O_2_ über dem Normwert (Normwert p_a_O_2_ bei Raumluft: p_a_O_2_ = 102 − 0,33 × Alter)

## Physiologische Grundlagen

Menschliche Zellen sind zur Aufrechterhaltung der zellulären Integrität und der zelltypuseigenen Funktionen von Sauerstoff zur Energiegewinnung abhängig. Eine Unterversorgung mit Sauerstoff führt zunächst zur anaeroben Energiegewinnung, die weitaus inneffizienter ist als die aerobe Energiegewinnung. Im weiteren Verlauf führt der Sauerstoffmangel zu einer unzureichenden Energieversorgung der Zellen mit schlussendlich Zellschäden oder Zelltod. Die Determinanten der Sauerstoffversorgung sind der Sauerstoffgehalt des Bluts (C_a_O_2_) und die Menge an Blut, die zu den Geweben gepumpt wird, der Cardiac Output (CO). Somit ergibt sich für das Sauerstoffangebot (DO_2_) die nachfolgende Formel, wobei S_a_O_2_ die Sauerstoffsättigung des Bluts und p_a_O_2_ den Anteil physikalisch gelösten Sauerstoffs im Blut bezeichnet:

$$\mathrm{DO}_{\mathrm{2}}\,=\,\mathrm{C}_{\mathrm{a}}\mathrm{O}_{\mathrm{2}}\,\times\,\mathrm{CO}$$

Der Sauerstoffgehalt des Bluts (C_a_O_2_) wird folgendermaßen errechnet (Hb = Hämoglobingehalt):

$$\mathrm{C}_{\mathrm{a}}\mathrm{O}_{\mathrm{2}}=\mathrm{Hb}\times \,\mathrm{SaO}_{\mathrm{2}}\,\times \,1{,}34 + (\mathrm{PaO}_{2} \times \,0{,}{003})$$

Damit ergibt sich folgende Formel für das Sauerstoffangebot (DO_2_):

$$\mathrm{DO}_{2}=\,\mathrm{CO}\times \mathrm{Hb}\times \,\mathrm{SaO}_{2}\,\times \,1{,}34\,+\,(\mathrm{PaO}_{2}\,\times \,0{,}003)$$

Somit lassen sich 4 beeinflussbare Variablen im Sauerstoffangebot identifizieren: Es ergibt sich 1. der Cardiac Output aus Schlagvolumen und Frequenz, beides Variablen, die physiologisch, durch Pathologien oder durch Medikamente in die eine oder andere Richtung beeinflusst werden können. Es besteht hier ein erster, auch therapeutisch beeinflussbarer Faktor, um das Sauerstoffangebot zu verändern.

Die 2. beeinflussbare Variable ist der Hämoglobingehalt des Bluts. Änderungen des Hämoglobingehalts können hier das DO_2_ in die eine oder andere Richtung verändern.

Als 3. geht die Sauerstoffsättigung des Hämoglobins in die Gleichung ein. Hier gilt ebenfalls eine direkte Beziehung zum Sauerstoffangebot. Auch diese Variable kann durch medizinische Intervention beeinflusst werden und ist eine wichtige Zielvariable bei der Sauerstofftherapie.

Diese bisherigen Variablen gehen alle als Multiplikatoren in die Gleichung ein, dies gilt jedoch nicht für die 4. Variable, den physikalisch gelösten Sauerstoff im Blut (p_a_O_2_), der nach Multiplikation mit dem Faktor 0,003 nur zum vorherigen Produkt addiert wird. Somit wird klar, dass die Relevanz des physikalisch gelösten Sauerstoffs am Anteil des Sauerstoffangebots minimal ist.

Die Relevanz des physikalisch gelösten Sauerstoffs am Anteil des Sauerstoffangebots ist minimal

**Figure 1:**
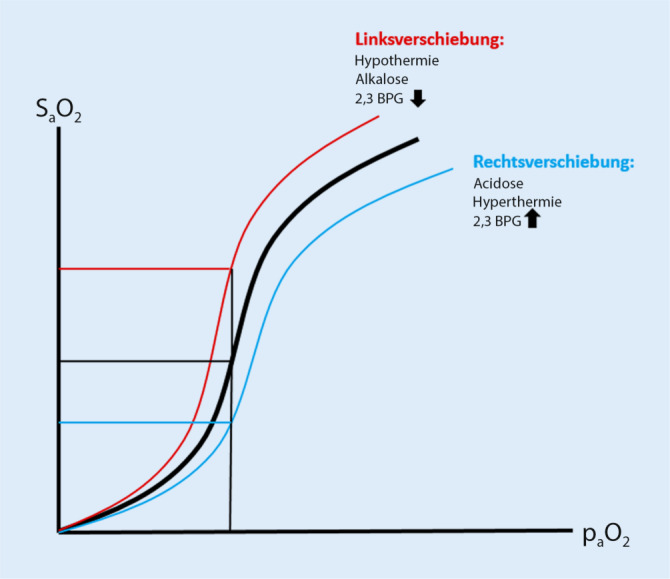

Auch der p_a_O_2_ lässt sich natürlich durch Interventionen wie Sauerstoffgabe oder Anpassung der Beatmungseinstellung verändern und korreliert im Rahmen der sigmoiden Sauerstoffbindungskurve eng mit der Sauerstoffsättigung. Ein p_a_O_2_ von 60 mm Hg entspricht etwa einer S_a_O_2_ von 90 %, ein p_a_O_2_ von 25 mm Hg etwa einer S_a_O_2_ von 50 %. Diese Korrelation ist aber abhängig von Temperatur, 2,3-Bisphosphoglycerat-Gehalt, pH-Wert und CO_2_-Gehalt des Bluts [1, 2]. Dadurch können bei gleichem p_a_O_2_ unterschiedliche S_a_O_2_-Werte zustande kommen (Abb. 1). Der Effekt der Stärke der Sauerstoffbindung an das Hämoglobin und damit auch die Abgabefähigkeit von O_2_ bei Verschiebungen der Sauerstoffbindungskurve nach links (O_2_ stärker an Hb gebunden) oder nach rechts (O_2_ schwächer an Hb gebunden) ist in den Empfehlungen von Sauerstoffzielparametern nicht berücksichtigt und hat auch in der Formel des DO_2_ keinen Einfluss.

Als Zielvariable der Sauerstofftherapie ist der p_a_O_2_ weniger sinnvoll, da er kaum einen Einfluss auf das DO_2_ hat und zudem nicht wie die S_p_O_2_ in nahezu Echtzeit gemessen werden kann. Allerdings erlaubt die Messung des p_a_O_2_ in gleichzeitiger Kenntnis des zugeführten Sauerstoffs die Abschätzung der Oxygenierungsleistung der Lunge. Hierzu existiert etwa den Horowitz-Index (HI), der den p_a_O_2_ ins Verhältnis zur inspiratorischen Sauerstoffkonzentration (F_i_O_2_) setzt: HI = p_a_O_2_ /F_i_O_2_ (0,21–1). Somit ist der p_a_O_2_ besser zur Beurteilung der Oxygenierungsleistung der Lunge als zur Therapiesteuerung geeignet.

Der p_a_O_2_ ist ein Monitoringparameter für die Oxygenierungsleistung der Lunge

Im klinischen Alltag ist immer wieder die Frage relevant, ob eine Modifikation der Beatmungseinstellungen aufgrund eines niedrigen p_a_O_2_ erfolgen sollte, um ein bestimmtes p_a_O_2_-Ziel zu erreichen. Unter der Annahme, dass eine Ziel‑S_a_O_2_ von 90 % festgelegt wurde, der gemessene p_a_O_2_ bei einer F_i_O_2_ von 50 % bei 65 mm Hg liegt und die SaO_2_ mit 93 % gemessen wird, liegt also die S_a_O_2_ als wesentliche Determinante der DO_2_ im Zielbereich. Der p_a_O_2_ gibt zwar Auskunft über die in diesem Fall wohl schlechte Oxygenierungsleistung der Lunge, muss aber aufgrund des minimalen Einflusses auf die DO_2_ bei gleichzeitig im Zielbereich liegender S_a_O_2_ nicht etwa durch die Erhöhung der F_i_O_2_ gesteigert werden. Damit ist der p_a_O_2_ also ein Monitoringparameter für die Oxygenierungsleistung der Lunge und S_a_O_2_ bzw. S_p_O_2_ sind Monitoringparameter in der Therapiesteuerung der Sauerstofftherapie.

## Physiologische Kompensationsmechanismen bei fallendem Sauerstoffangebot

Kommt es im Oxygenierungssystem des Körpers zu Problemen und fällt damit das DO_2_ ab, gibt es akute Kompensationsmechanismen, um die Sauerstoffversorgung der Zellen aufrechtzuerhalten. Anders ausgedrückt bleibt die Sauerstoffaufnahme (VO_2_) des Körpers bzw. der Organe auch bei sinkendem Sauerstoffangebot (DO_2_) über einen relativ weiten Bereich konstant, bevor bei weiter fallendem DO_2_ auch die VO_2_ und damit die Sauerstoffversorgung der Zellen abfällt und die VO_2_ abhängig vom DO_2_ wird.

Bei gesunden Individuen besteht über weite Strecken keine Abhängigkeit der VO_2_ von der DO_2_

Dies trifft zunächst auf gesunde Individuen zu, bei denen die Kompensationsmechanismen physiologisch funktionieren. Hierzu führten Shibutani et al. [3] an narkotisierten gesunden Probanden eine Untersuchung durch. Es wurde gezeigt, dass es erst bei einem DO_2_ <330 ml/min × m^2^ zu einem Abfall der VO_2_ kommt und es über dieser Grenze bei einer weitgehend konstanten VO_2_ trotz fallender DO_2_ bleibt. Anders ausgedrückt besteht bei gesunden Individuen über weite Strecken keine Abhängigkeit der VO_2_ von der DO_2_, da der Körper in diesem Bereich über Kompensationsmechanismen verfügt, die verhindern, dass es bei abnehmendem Sauerstoffangebot zu einer Organhypoxie kommt.

Ein akuter Kompensationsmechanismus besteht hier in einer Steigerung der Sauerstoffextraktionsrate. Diese Sauerstoffextraktionsrate liegt physiologischer Weise und in Ruhe bei etwa 15–30 %, d. h.: Vom arteriellen Sauerstoffgehalt „extrahieren“ die Körperzellen in Summe etwa 20–30 % des enthaltenen Sauerstoffs. Hier haben die verschiedenen Organe unterschiedliche Extraktionsraten: das Herz etwa 60 %, die Leber etwa 50 % und die Nieren etwa 15 %. In Summe aller Organsysteme ergeben sich 15–30 %. Kommt es nun zu einem Abfall des DO_2_ durch Verminderung einer oder mehrerer der Determinanten des DO_2_ oder steigt die VO_2_ überproportional zum DO_2_ an (wie z. B. bei körperlicher Belastung), steigt auch die Sauerstoffextraktionsrate an. Diese kann Werte bis zu 70 % bei maximaler körperlicher Belastung annehmen. Zur Berechnung der Sauerstoffextraktionsrate (O_2_ER) werden eine arterielle und eine gemischt-venöse Blutprobe benötigt. Die gemischt-venöse Probe enthält auch das desoxygenierte Blut des Herzens. Alternativ zur gemischt-venösen Probe kann auch Blut aus einem zentralen Venenkatheter (ZVK), idealerweise mit Lage der ZVK-Spitze im rechten Vorhof, verwendet werden. Der Messfehler ist hierbei tolerabel.

Die Berechnung lautet dann:

$$\mathrm{O}_{2}\text{ER in }\mathrm{\% }=[(\mathrm{SaO}_{2}2 - \mathrm{SvO}_{2})/\mathrm{SaO}_{2}]\times 100$$

Hierbei gibt S_v_O_2_ die gemischt-venöse Sauerstoffsättigung an.

Ein weiterer Kompensationsmechanismus bei sinkendem DO_2_ ist die Steigerung des Herzzeitvolumens, sofern das kardiovaskuläre System dazu in der Lage ist. Exemplarisch wäre auch hier wieder die körperliche Anstrengung zu sehen, bei der es neben der bereits beschriebenen Steigerung der Sauerstoffextraktionsrate auch zu einer Steigerung des Herzzeitvolumens kommt, um dem erhöhten Sauerstoffbedarf der Muskulatur und damit der steigenden Sauerstoffaufnahme VO_2_ gerecht zu werden. Zusätzlich spielen sich Kompensationsmechanismen auf mikrovaskulärer Ebene ab. Es kommt hier zu einer Steigerung des mikrovaskulären Blutflusses und damit zu einer Steigerung des regionalen Sauerstoffangebots [4].

Die Steigerung des Herzzeitvolumens ist ein Kompensationsmechanismus bei sinkendem DO_2_

Die bei abfallendem Sauerstoffpartialdruck, etwa in großer Höhe, oder bei Hypoxämie auftretende Hyperventilation führt neben einer Linksverschiebung der Sauerstoffbindungskurve durch die respiratorische Alkalose dazu, dass der alveoläre CO_2_-Partialdruck abfällt. Nachdem die Summe der Partialdrücke aller Gase in den Alveolen konstant ist, wie durch die Alveolargasgleichung definiert (p_A_O_2_ = F_i_O_2_ × (p_atm_-p_H2O_) − p_a_CO_2_/RQ; RQ = respiratorischer Quotient), nimmt durch die Hyperventilation der alveoläre CO_2_-Partialdruck ab und der alveoläre Sauerstoffpartialdruck steigt an, was zu einer Steigerung des p_a_O_2_ führen kann.

Zu bedenken gilt, dass alle diese akuten Kompensationsmechanismen bei kranken oder kritisch kranken Patienten nur eingeschränkt oder gar nicht funktionieren und somit die Toleranz für ein fallendes DO_2_ geringer sein kann.

## Marker einer Hypoxämie

Zwar können bei Einzelorgansystemen, wie Herz, Leber oder Nieren, durch Anstieg von organspezifischen Laborwerten Dysfunktionen erkannt werden, diese müssen aber nicht primär durch ein zu geringes Sauerstoffangebot verursacht werden. Hier bieten sich andere globalere Messwerte an, die zwar auch nicht ideal sind, um einen globalen Sauerstoffmangel beim Intensivpatienten zu erkennen, die aber im Gesamtbild der Beurteilung, ob ein ausreichendes Sauerstoffangebot vorliegt, wichtige Bausteine sein können.

### Laktat

Historisch wurde Laktat immer mit anaerobem Stoffwechsel gleichgesetzt, was es per se zu einem idealen Marker für eine Sauerstoffunterversorgung des Körpers machen würde. Laktat kann aber auch auf andere Weise unter aeroben Bedingungen gebildet werden.

Zu einem Laktatanstieg kann es allerdings auch unter verschiedenen anderen, auch aeroben, Bedingungen kommen. Diese sog. Typ-B-Laktatämie kommt etwa bei gesteigertem Pyruvatanfall durch vermehrte Glykolyse, durch etwa endotoxinvermittelte Mitochodriendysfunktion oder eine durch Thiaminmangel bedingte Dysfunktion der Pyruvatdehydrogenase, die für die Aufnahme von Pyruvat in die Mitochondrien notwendig ist, vor.

Medikamente, Toxine oder hämatologische Erkrankungen können zur Laktatämie führen

Auch die endogen katecholaminbedingte Glykolyse und die Aktivierung der Na-K-ATPase bei Sepsis führen unter aeroben Bedingungen zu einem Laktatanstieg. Gleichzeitig kann es bei entsprechenden Organdysfunktionen zu einer verminderten hepatischen oder renalen Laktatclearance kommen. Auch Medikamente, Toxine oder hämatologische Erkrankungen können zur Laktatämie unter aeroben Bedingungen führen [5].

Nachdem auch eine verminderte Mikrozirkulation zu einer regionalen Hypoxie führen kann, sollte bei Verwendung von Laktat als Marker für eine DO_2_-abhängige VO_2_ – also eine globale Hypoxie – zunächst versucht werden, die Mikrozirkulation zu verbessern. Im Wissen um den Ursprung und die Differenzialdiagnosen einer Laktatämie kann Laktat ein wertvoller Marker sein, um ein kritisches Sauerstoffangebot zu erkennen.

### Sauerstoffextraktionsrate

Ein weiterer Parameter zur Beurteilung, ob ein kritisches DO_2_ vorliegt, ist die bereits erwähnte Sauerstoffextraktionsrate (O_2_ER). Analog trifft dies auf die zentralvenöse Sättigung (S_c_VO_2_) zu, die ebenfalls ein Surrogatparameter zur Beurteilung der Balance zwischen DO_2_ und VO_2_ ist. Somit ist bei einer O_2_ER < 30 % bzw. einer ScVO_2_ > 65 % von einem nichtkritischen DO_2_ auszugehen.

Allerdings gibt es auch hier Fehlerquellen, die beachtet werden müssen. Die Erkenntnis, dass es Patienten, insbesondere mit Sepsis, gibt, bei denen eine normale oder hochnormale zentralvenöse Sättigung mit einer erhöhten Mortalität einhergeht [6], hat gezeigt, dass die Sauerstoffaufnahme aus dem Blut in die Zellen aufgrund von Zellmembranödem, anderweitig verlängerter Diffusionsstrecke, Beeinträchtigung der mitochondrialen Funktion oder globalen bzw. regionalen Mikrozirkulationsstörungen gestört sein kann und die globale Sauerstoffextraktion trotz Gewebehypoxie nicht steigt. Somit ist die O_2_ER bzw. S_c_VO_2_ insbesondere bei Patienten mit schwerer Sepsis kritisch zu sehen, kann aber bei isoliertem respiratorischem Versagen bzw. im kurzfristigen Verlauf durchaus zur Abschätzung eines grenzwertigen oder kritischen DO_2_ herangezogen werden.

## Hypoxietoleranz

Bahr et al. [7] zeigten, dass Patienten, die aufgrund eines akuten Lungenversagens (ARDS) eine extrakorporale Membranoxygenierung (ECMO) benötigten und während der ECMO eine im Mittel niedrige S_p_O_2_ (89 %) hatten, Jahre später im Vergleich zu der Gruppe von Patienten, die unter ECMO normale SpO_2_-Werte erreichten (97 %), kein neurokognitives Defizit aufwiesen. Die Laktatwerte waren bei diesen hypoxämischen Patienten normal und die Präoxygenatorsättigung, also die Sättigung des venösen Bluts nach der Sauerstoffextraktion durch den Körper (entsprechend der S_C_VO_2_), betrug im Mittel 75 %.

Eine längerfristige Hypoxämie unter ECMO führte nicht zu neurokognitiven Schäden

In einer Vorgängerstudie zeigten Holzgraefe et al. bei 7 ECMO-Patienten mit noch geringeren SpO_2_-Werten (80 %) ebenfalls, dass eine längerfristige Hypoxämie (ECMO-Dauer bis zu 349 Tage) nicht zu neurokognitiven Schäden führte. Auch diese Patientengruppe hatte normale Laktatwerte (Mittelwert 1,4 mmol/l) und eine normale zentralvenöse Sättigung (Mittelwert 74,4 %).

Grocott et al. führten Im Jahr 2009 Blutgasanalysen bei Höhenbergsteigern am Mount Everest in einer Höhe von 8400 m durch. Der mittlere p_a_O_2_ betrug 24,6 mm Hg und die mittlere S_a_O_2_ betrug 54 %. Die mittlere Laktatkonzentration war mit 2,2 mmol/l nur leicht erhöht. Auch im Mount-Everest-Basislager auf 5300 m Höhe, wo sich die Bergsteiger teilweise wochenlang aufhalten, beträgt die mittlere SpO_2_ nur 80 %. Natürlich waren diese Bergsteiger durch längere Akklimatisierungszeit im Basislager adaptiert, so betrug etwa der mittlere Hb-Wert bei den Probanden in der Grocott-Studie 19,3 mg/dl. Somit gibt es Hinweise für eine beträchtliche Hypoxämietoleranz des menschlichen Körpers.

## Happy oder Silent Hypoxämie

In der Pandemie die Coronaviruserkrankung 2019 (COVID-19) hat sich der Begriff der Happy/Silent Hypoxämie geprägt. Darunter versteht man Patienten, die trotz einer niedrigen Sauerstoffsättigung kaum das Symptom Dyspnoe aufweisen. Zwar tritt dieses Phänomen bei COVID-19 gehäuft auf, jedoch gibt und gab es solche Patienten auch schon vor dem Auftreten von COVID-19. Eine Erklärung dafür liefert die zugrunde liegende Pathophysiologie.

Dyspnoe ist ein Symptom und speziell hier ist es wichtig, zwischen Symptomen (= subjektive Wahrnehmungen) und Zeichen (= objektiv messbare Parameter) zu unterscheiden. Bei Dyspnoe muss auch die Tachypnoe (= schnelle Atemfrequenz) von der Hyperpnoe (= tiefere Atemzüge) unterschieden werden.

Dyspnoe wird durch erhöhte Atemarbeit hervorgerufen

Um das Symptom Dyspnoe hervorzurufen, sind im Wesentlichen folgende pathophysiologischen Veränderungen ursächlich: Zunächst wird Dyspnoe durch erhöhte Atemarbeit hervorgerufen. Hierfür sind unter anderem die Aktivierung von Mechanorezeptoren in Lunge und Thoraxwand und die Aktivierung bzw. Erschöpfung der Atemmuskulatur verantwortlich. Der primäre Trigger, der zu einer Steigerung des Atemantriebs (ist noch keine Dyspnoe) führt, ist eine Erhöhung des p_a_CO_2_ (Hyperkapnie), die über PH-Wertveränderungen an den zentralen und peripheren Chemorezeptoren den Atemantrieb und damit die Ventilation erhöht. Pulmonale Gründe für eine Hyperkapnie können ein vergrößerter Totraum, auch auf alveolarer Ebene, eine verminderte Lungencompliance oder ein erhöhter Atemwiderstand, wie z. B. bei Bronchokonstriktion, sein.

Eine isolierte Hypoxämie bei nichterschöpfter Atemmuskulatur führt nicht zum Symptom Dyspnoe

Eine isolierte Hypoxämie führt kaum zum Symptom Dyspnoe, solange die Atemmuskulatur durch Hyperventilation nicht erschöpft ist. Somit können sich Pathologien, die bei normaler Lungencompliance, normalem Totraum und normalem Atemwegswiderstand ohne anderweitig bestehender Hyperkapnie zu einer Hypoxämie führen, ohne wesentliche Dyspnoe präsentieren, solange die Atemarbeit nicht erhöht ist. Bei COVID-19-Patienten scheint es durch vaskuläre Veränderungen zu einem ausgeprägten pulmonalen Shunt zu kommen. Dieser Shunt führt bei noch ausreichender Decarboxylierungsleistung aufgrund der normalen Lungencompliance und der normalen Atemwegswiderstände zwar zu einer Hypoxämie, jedoch nicht zwangsläufig zu Dyspnoe, solange die Atemmuskulatur die notwendige Leistung erbringen kann. Zusätzlich werden direkte Einflüsse des Virus auf die Empfindlichkeit der Chemorezeptoren diskutiert [8].

Die Shuntphysiologie der Happy Hypoxämie bei COVID-19 kann aber auch bei Patienten mit anderer Pathologie zutreffen. Das einfachste Beispiel hier wäre ein Patient mit intrakardialem Rechts-Links-Shunt, der ebenfalls eine Hypoxämie, jedoch keine erhöhte Atemarbeit und damit keine Dyspnoe aufweisen muss. Auch ein intrapulmonaler Shunt, etwa durch eine Atelektase, kann bei erhaltender Lungencompliance und normalen Atemwegswiderständen zu einer Hypoxämie ohne Dyspnoe führen.

## Leitlinien und Studien

Welche Sauerstoffwerte sollen nun in Anbetracht des bisher diskutierten Inhalte angestrebt werden? Die aktuellen Leitlinien geben relativ ähnliche Zielwerte an. Die Empfehlung des *ARDSnet* für Patienten mit einem ARDS nennen einen p_a_O_2_ von 55–80 mm Hg bzw. eine SpO_2_ von 88–95 % [9]. Die* Surviving Sepsis Campaign Guidelines on the Management of Adults with Coronavirus Disease 2019 (COVID-19) in the ICU* empfehlen eine Sauerstofftherapie bei einer SpO_2_ < 90 % [10]. Die deutsche S3-Leitlinie *Empfehlungen zur stationären Therapie von Patienten mit COVID-19* empfiehlt eine S_P_O_2_ von > 92 % (bei Patienten mit chronisch-obstruktiver Lungenerkrankung [COPD] > 88 %; [11]). Die deutsche S3-Leitlinie *Sauerstoff in der Akuttherapie beim Erwachsenen* empfiehlt eine Ziel‑S_p_O_2_ von 92–96 %. Bei Patienten mit vorbestehender COPD oder einem anderweitigen Hyperkapnierisiko wird eine Ziel‑S_p_O_2_ von 88–92 % angegeben [12].

Die deutsche S3-Leitlinie empfiehlt eine Ziel‑S_p_O_2_ von 92–96 %

Die Evidenz dieser Empfehlungen leitet sich aus wenigen Studien mit relativ geringen Patientenzahlen gemischter Kollektive auf der Intensivstation (ICU) ab, die hauptsächlich S_p_O_2_-Werte im Bereich von 90 % mit Werten über 96 % verglichen. Die primäre Intention vieler dieser Studien war zu klären, ob höhere Sauerstoffwerte schädlich sind und nicht wie tief tolerable S_p_O_2_-Werte liegen.

Die rezenteste und auch größte dieser Studien war der HOTICU Trial, bei dem multizentrisch an 2928 Patienten eines gemischten Patientenkollektivs auf der ICU untersucht wurde, ob ein liberales Sauerstoffziel mit einem p_a_O_2_ von 60 mm Hg vs. ein konservatives Sauerstoffziel mit einem p_a_O_2_ von 90 mm Hg einen Unterschied in der Mortalität verursacht. Es konnte hier kein Unterschied in der 90-Tage-Mortalität gezeigt werden. Auch bei den sekundären Endpunkten, wie Schlaganfall, Myokardinfarkt oder intestinale Ischämie, gab es keinen Unterschied. Im Endeffekt lag der mittlere p_a_O_2_-Wert in der liberalen Gruppe bei 70,8 mm Hg und die SpO_2_ bei 93 %; in der konservativen Gruppe lag der p_a_O_2_ bei 93,9 mm Hg, die SpO_2_ bei 96 %.

Keine der Studien untersuchte Sauerstoffsättigungen < 88 %

In der LOCO_2_-Studie [13] wurde eine S_p_O_2_ > 96 % mit einer S_p_O_2_ 88–92 % verglichen. Diese Studie wurde aus Sicherheitsbedenken nach 201 Patienten abgebrochen, da es in der Gruppe mit dem niedrigeren Sättigungsziel mehr Todesfälle und 5 Mesenterialischämien gab. Weitere Studien mit ähnlichem Design, aber ohne signifikant schlechteres Outcome für eine konservativere Sauerstoffsättigungszielsetzung waren ICU-ROX [14] oder OXYGEN-ICU [15]. Keine dieser Studien hatte jedoch das Ziel, Sauerstoffsättigungen < 88 % zu untersuchen.

Diese Studien liefern also keine wirkliche Antwort auf die Frage, ob ein deutlich niedrigeres Oxygenierungsziel bzw. die Toleranz deutlich niedrigerer Oxygenierungswerte tatsächlich mit einer höheren Mortalität einhergehen.

Eine Ausnahme zu den bereits angeführten Empfehlungen stellt die Leitlinie der Extracorporeal Life Support Organization (ELSO) für die ECMO-Therapie [16] dar. Dort wird bei einer venovenösen ECMO eine S_a_O_2_ > 80 % und eine venöse-arterielle Sättigungsdifferenz (entsprechend der Sauerstoffextraktionsrate) von 20–30 % empfohlen.

Hinsichtlich der Frage, ob der inzwischen häufig angewandte Weg, COVID-19-Patienten eher später zu intubieren und gegebenenfalls eine gewisse Hyoxämie zu tolerieren, sicher ist, zeigten Papoutsi et al. in einer rezenten Metaanalyse [17] mit fast 9000 Patienten, dass eine spätere Intubation nicht mit einem schlechteren Outcome einhergeht. Somit scheint bei diesen Patienten möglicherweise eine gewisse Toleranz von Hypoxämie gerechtfertigt zu sein, sofern die Surrogatparameter der Gewebeoxygenierung – wie bereits beschrieben – sowie die Atemarbeit und damit die Dyspnoesymptomatik innerhalb tolerabler Grenzen liegen. Allerdings werden auch hier keine eindeutigen Sauerstoffgrenzwerte herausgearbeitet bzw. bleibt unklar, ob und wie lange bei den später intubierten Patienten eine Hypoxämie bestand.

## Hyperoxämie

Neben den negativen Effekten einer Hypoxämie ist inzwischen aber auch eindeutig bewiesen, dass eine Hyperoxämie in verschiedenen Situationen negative Auswirkungen hat. In einer Metaanalyse mit 25 Studien und über 16.000 Intensivpatienten legten Chu et al. dar, dass für verschiedene Erkrankungen, wie Sepsis, Apoplex, Trauma, Myokardinfarkt, Kreislaufstillstand oder Notfallchirurgie, eine liberale Sauerstofftherapie mit S_p_O_2_-Werten > 96 % mit einem schlechteren Outcome vergesellschaftet ist. Somit sollte nicht nur die Vermeidung einer Hypoxämie, sondern ebenso die Vermeidung einer Hyperoxämie im klinischen Alltag angestrebt werden [18, 19].

## Fazit für die Praxis

- Bei nichtrelevant chronisch-pulmonal vorerkrankten Patienten ist eine Sauerstofftherapie zur Anhebung der Sauerstoffsättigung auf Werte von 88–96 % durchzuführen, eine Hyperoxämie jedoch zu vermeiden.
- Muss eine extrakorporale Membranoxygenierung (ECMO) durchführt werden, kann eine niedrigere Sauerstoffsättigung von bis zu 80 % toleriert werden.
- Ist eine ECMO keine Therapieoption, gilt es abzuschätzen, ob eine Steigerung der Therapieinvasivität zugunsten der Zielsauerstoffsättigung (Ziel-SpO_2_) gerechtfertigt ist. Abseits von Grenz- oder Extremsituationen besteht aktuell jedoch keine Evidenz für den Nutzen oder die Ungefährlichkeit eine permissive Hypoxämie mit SpO_2_-Werten < 88–92 %.
- Bei Patienten mit Coronaviruserkrankung 2019 (COVID-19), die eine Hypoxämie klinisch relativ gut tolerieren, kann mit einer Intubation zunächst gewartet werden. Aufgrund fehlender Studien zum Zusammenhang von Hypoxämiegrad und Organschäden muss die Entscheidung zur Intubation letztendlich aufgrund klinischer Parameter ohne wirkliche Evidenz getroffen werden.
